# Physical inactivity prevalence and trends among Mexican adults: results from the National Health and Nutrition Survey (ENSANUT) 2006 and 2012

**DOI:** 10.1186/1471-2458-13-1063

**Published:** 2013-11-11

**Authors:** Catalina Medina, Ian Janssen, Ismael Campos, Simón Barquera

**Affiliations:** 1 Instituto Nacional de Salud Pública, Av. Universidad 655, Col. Sta. María Ahuacatitlan, 62100 Cuernavaca, Morelos, Mexico; 2 School of Kinesiology and Health Studies, Queen’s University, Kingston, Ontario, Canada; 3 Department of Public Health Sciences, Queen’s University, Ontario, Canada

**Keywords:** Physical inactivity, IPAQ, Cross-sectional studies, Mexico, National surveys

## Abstract

**Background:**

Lifestyles such as unhealthy diets and the lack of physical activity have been contributed to the increased prevalence of obesity. In 2012, the world health organization published the first global recommendation for physical activity and health. People who do not meet at least 150 minutes of moderate-to-vigorous physical activity are considered to be physically inactive. The prevalence of physical inactivity worldwide is 31%, however there is insufficient data from prevalence and trends of physical inactivity in Mexican population. The purposes of this study are to describe the physical inactivity prevalence and recent trends in Mexican adults and to examine the association between physical inactivity with biologic and sociodemographic characteristics.

**Methods:**

Representative samples of 17,183 and 10,729 adults (aged 20 to 69 years) who participated in the National Health and Nutrition Survey (ENSANUT) in 2006 and 2012, respectively. Moderate-to-vigorous physical activity (MVPA) was assessed using the short form version of the International Physical Activity Questionnaire (IPAQ), which was administered in face-to-face interviews. Self-reported IPAQ MVPA levels were adjusted using an equation derived from a previous validation study. Participants were considered inactive if they engaged in <150-minutes/week of moderate physical activity or <75 minutes/week of vigorous physical activity according to WHO classification criteria.

**Results:**

The prevalence of physical inactivity was significantly higher in 2012 (19.4%, 95% CI: 18.1, 20.7) than in 2006 (13.4%, 95% CI: 12.5, 14.5). Adults in the obese category, 60–69 age group, and those in the highest socioeconomic status tertile were more likely to be physically inactive.

**Conclusions:**

The proportion of the Mexican adult population who do not meet the minimum WHO physical activity criteria has increased by 6% points between 2006 and 2012. Given the increasing prevalence of obesity, the aging of the population, and the shift in socioeconomic status in Mexico, physical inactivity could continue to increase in the coming years unless effective public health interventions are implemented.

## Background

Lifestyle changes such as decreasing physical activity levels and unhealthy diets have contributed to the rise in obesity and non-communicable diseases [1]. Physical inactivity causes 5.3 million annual deaths worldwide and 6-10% of deaths caused by non-communicable diseases are attributed to physical inactivity [2]. Currently Mexico is facing an epidemic of non-communicable chronic diseases with increasing prevalence of obesity. Physical inactivity is one of the major factors contributing to this epidemic and one of the main risk factors for mortality in Mexico [3].

In 2010 the World Health Organization published the first global recommendations for physical activity and health [4]. These recommendations indicate that to obtain health benefits, adults need to accumulate, in bouts of at least 10 minutes, a minimum of 150 minutes per week of moderate physical activity or 75 minutes of vigorous physical activity or their respective combination [4]. People who do not meet this recommended volume of moderate-to-vigorous physical activity (MVPA) are considered to be physically inactive. Recent estimates indicate that the worldwide prevalence of physical inactivity in adults is 31% [1,5].

The international physical activity questionnaire (IPAQ) can be used to assess adherence to the WHO physical activity recommendations [6]. The short form version of the IPAQ was included within the Mexican National Health and Nutrition Survey (ENSANUT) in 2006 and 2012. The objectives of this article are to describe the current prevalence of physical inactivity in Mexican adults (20–69 year olds), to examine changes in the prevalence of physical inactivity over the past 6 years, and to examine the association between physical inactivity and several sociodemographic and biological characteristics such as gender, age, body mass index (BMI) status, location of residence, and socioeconomic status (SES).

## Methods

### Design and participants

The ENSANUT used a probabilistic multistage stratified cluster sampling design. The sample size was designed to be representative of the country and regions, and their expansion factors and sampling weights were used in all analyses to render nationally representative estimates [7]. The surveys were conducted between October and May in 2005–2006 and 2011–2012. In 2006 data from 36,170 households was collected to obtain a sample of approximately 69,000 individuals. In 2012 data from 50,528 households was collected to obtain a sample of 89,000. In 2006, physical activity data were obtained on 17,183 adults who represented 55,165,527 people. In 2012, physical activity data were obtained from 10,729 adults who represented 65,252,418 people. Adults were defined as those between 20–69 years old according to the ENSANUT methodology. Detailed descriptions of the ENSANUT methodology are published elsewhere [8,9]. All participants provided informed consent prior to participating. The National Public Health Institute Ethics Review Board of Mexico approved the study protocol.

### Physical activity assessment using the international physical activity questionnaire

To assess physical activity, the Spanish version of the short form IPAQ was applied by trained personnel in face-to-face interviews. In 2006, the questionnaire was collected using a paper and pencil-based interview while a computer-based interview was used in 2012. The IPAQ assess the amount of moderate (3–5.9 metabolic equivalents (METs)), and vigorous (≥6 METs) physical activity accumulated in bouts of at least 10 minutes over the previous 7 days. Validity and reliability results from the short form IPAQ have been obtained in several countries, including Mexico [6,10,11].

The IPAQ questionnaire data was cleaned using established IPAQ protocols [12]. Based on responses obtained from the IPAQ, minutes per week of moderate physical activity (including walking) and vigorous physical activity were calculated for each participant. Moderate physical activity values were added to vigorous physical activity values to obtain MVPA minutes per week. Hereafter, we refer to the original MVPA data collected from the IPAQ as the “unadjusted” MVPA data.

### Classification into WHO physical activity categories

Total minutes per week spent in MVPA was used to classified participants into three WHO physical activity categories [4]: “*physically inactive*” if they participated in <150 min/week of moderate intensity, or <75 min/week of vigorous intensity, or an equivalent combination of the two intensities, “*sufficiently active*” if they had 150–299 min/week of moderate intensity, or 75–149 min/week of vigorous intensity, or an equivalent combination, and “*very active*” if they participated in ≥300 min/week of moderate intensity, or ≥150 min/week of vigorous intensity, or an equivalent combination, considering bouts of at least 10 minutes. Participants were classified as very active if they accumulated at least twice the minimum WHO recommendation.

To assign participants to the appropriate physical activity category we multiplied their vigorous intensity minutes by two and added it to their minutes of moderate physical activity. We then used the moderate activity cut-points (<150 min/week for the physically inactive category, 150–299 min/week for the sufficiently active category and ≥300 min/week for the very active category) to determine the appropriate physical activity category.

### Adjusting the international physical activity questionnaire data

The IPAQ and other self-reported questionnaire measures substantially over-report MVPA levels by comparison to those obtained objectively using accelerometers and other electronic movement sensing devices [13-17]. Recent physical inactivity surveillance studies have adjusted the over-reported questionnaire data, including those obtained using the IPAQ, so that they more closely reflect the truth [1]. To adjust MVPA for over-reporting, we developed an equation based in a validity study recently conducted by our group [11]. To achieve this, we obtained IPAQ and objectively measured MVPA on a sample of Mexican adults (19–69 years old) using accelerometers. Standardized protocols were used to verify the completeness of the accelerometry data, to clean it, and to determine the weekly volume of moderate (3–5.9 METs) and vigorous (≥6 METs) physical activity completed in bouts of at least 10 minutes [11]. MVPA was obtained by adding moderate physical activity (including walking) minutes per week plus vigorous physical activity minutes per week multiplied by two. With this information we developed the following equation to predict the objective MVPA with the estimated MVPA by IPAQ, and used it to adjust MVPA from ENSANUT 2006 and 2012:

$$\begin{array}{c} \text{Adjusted}\;\mathrm{MVPA}=10.8335\surd \mathrm{IPAQ}\;\mathrm{MVPA}\;\left(\text{minutes}\;\text{per}\;\text{week}\right)*\\ {}*\text{Moderate}\;\left(\text{including}\;\text{walking}\right),\text{plus}\;\left(\text{vigorous}\right)\;2 \end{array}$$

Adjusted MVPA minutes per week were then classified using the moderate activity cut-point previously mentioned.

### Sociodemographic and biological variables

#### *Geographic areas*

Differences in MVPA were considered based on whether participants lived in an urban (≥2, 500 residents) or rural (<2,500 residents) area and based on the region of the country they lived in. ENSANUT is representative of four geographic areas of the country including: *North* (Baja California, Southern Baja California, Coahuila, Durango, Nuevo Leon, Sonora, Sinaloa, Tamaulipas and Zacatecas), *Central* (Aguascalientes, Colima, Guanajuato, Hidalgo, Jalisco, Mexico (excepting D.F. and metropolitan areas), Michoacan, Nayarit, Querétaro, San Luis Potosi and Tlaxcala), *Distrito Federal* (D.F. and metropolitan areas), and *South* (Campeche, Chiapas, Guerrero, Morelos, Oaxaca, Puebla, Quintana Roo, Tabasco, Veracruz and Yucatan).

#### *Socioeconomic status and education (SES)*

A SES index was previously constructed and validated by the Center of Survey Research at the Mexican National Institute of Public Health [18] by combining 8 variables that assessed the household properties and available services including: construction materials of the floor, ceiling, and walls; sleeping rooms; water accessibility; vehicle ownership; household goods (refrigerator, washing machine, microwave, stove, boiler); and electrical goods (television, radio, telephone, and computer). The index was divided into tertiles and used as a proxy for low, medium, and high SES. Education level was stratified into three groups according to the highest level of education obtained: primary or less, secondary, and high school or higher [18].

#### *Anthropometric measurements*

Weight and height were measured to the nearest 0.1 kg and 0.1 cm, and the BMI was calculated as kg/m^2^. BMI status was based upon the WHO endorsed adult cut-points as: underweight (<18.5 kg/m^2^), normal weight (18.5-24.9 kg/m^2^), overweight (25.0-29.9 kg/m^2^), or obese (≥30.0 kg/m^2^) [19].

### Statistical analysis

The sample design characteristics (sample weights, cluster and strata variables) were taken into consideration for all analyses. Means, interquartile ranges, and percentages were used to describe the MVPA levels (minutes/week), physical activity categories, and sociodemographic characteristics of the participants. Physical activity was described within the entire sample and within strata based on the sociodemographic characteristics. Kernel-density plots were used to compare the proportional amount of minutes per week of MVPA by survey (2006 vs. 2012) and a general linear model was used to compare minutes per week of MVPA by biological and sociodemographic characteristics. Bivariate logistic regression models were used to evaluate the association between physical inactivity and biological and sociodemographic variables. This was followed by a single multivariate model that simultaneously included all of the biological and sociodemographic variables.

## Results

Descriptive information on the 20–69 year olds adults who completed the IPAQ in ENSANUT 2006 and 2012 are provided in Table 1. Data were available for 17,175 adults in 2006 and 10,591 adults in 2012.

**Table 1:** Sociodemographic and anthropometrics characteristics in adults of 20–69 year old Mexican adults, ENSANUT 2006 and ENSANUT 2012

	**2006**			**2012**		
	**n (17,175)**	**N (55,137,118)**	**% (95% CI)**	**n (10,591)**	**N (64,205,112)**	**% (95% CI)**
**Gender**						
Men	6,356	25,434,379	46.1 (44.8,47.5)	4,260	30,042,693	46.8 (45.0,48.6)
Women	10,819	29,702,739	53.9 (52.5,55.2)	6,331	34,162,419	53.2 (51.4,55.0)
**Age group**						
20-29	3,784	15,662,567	28.4 (27.3,29.5)	2,373	18,355,767	28.6 (26.8,30.5)
30-39	5,246	14,409,428	26.1 (25.1,27.2)	2,844	16,173,449	25.2 (23.6,26.9)
40-49	3,948	11,815,191	21.4 (20.5,22.4)	2,320	13,295,192	20.7 (19.5,22.0)
50-59	2,450	7,941,781	14.4 (13.6,15.3)	1,697	10,050,816	15.7 (14.5,16.9)
60-69	1,747	5,308,150	9.6 (8.9,10.4)	1,357	6,329,888	9.9 (8.9,10.9)
**BMI Classification**						
Normal weight	4,422	15,154,351	30.0 (28.7,31.3)	2,686	17,925,934	29.3 (27.4,31.2)
Overweight	6,325	20,356,225	40.3 (39.0,41.6)	3,846	22,622,942	36.9 (35.2,38.7)
Obese	5,131	14,997,219	29.7 (28.6,30.9)	3,557	20,704,455	33.8 (32.1,35.6)
**Region of country**						
South	6,756	16,444,862	29.8 (26.6,33.2)	3,722	19,558,726	30.5 (29.0,31.9)
Central	6,452	16,669,503	30.2 (27.0,33.7)	3,714	19,012,496	29.6 (28.2,31.1)
North	3,249	11,037,504	20.0 (17.6,22.7)	2,820	12,871,379	20.0 (19.0,21.1)
D.F. and metropolitan area	718	10,985,247	19.9 (16.0,24.5)	335	12,762,512	19.9 (17.9,22.1)
**Rural/urban areas**						
Rural	7,113	17,382,281	31.5 (28.0,35.2)	3,805	15,699,938	24.5 (23.1,25.8)
Urban	10,062	37,754,837	68.5 (64.8,72.0)	6,786	48,505,174	75.5 (74.2,76.9)
**Socioeconomic status**						
Low	8,408	21,184,496	38.6 (36.0,41.3)	3,799	17,422,714	27.1 (25.6,28.7)
Medium	5,457	17,781,380	32.4 (30.8,34.1)	3,620	20,509,353	31.9 (30.2,33.8)
High	3,256	15,928,267	29.0 (26.8,31.3)	3,172	26,273,045	40.9 (38.9,43.0)
**Education level**						
Primary or less	8,625	24,568,355	49.7 (47.8,51.7)	2,880	13,572,984	21.1 (19.7,22.7)
Secondary	3,794	12,775,687	25.8 (24.5,27.2)	5,341	32,337,711	50.4 (48.4,52.3)
High school or higher	2,746	12,078,632	24.4 (22.7,26.3)	2,370	18,294,417	28.5 (26.6,30.4)

n = sample size; N = weighted sample size; % (95% CI) = prevalence (95% confidence interval).

**Figure 1:**
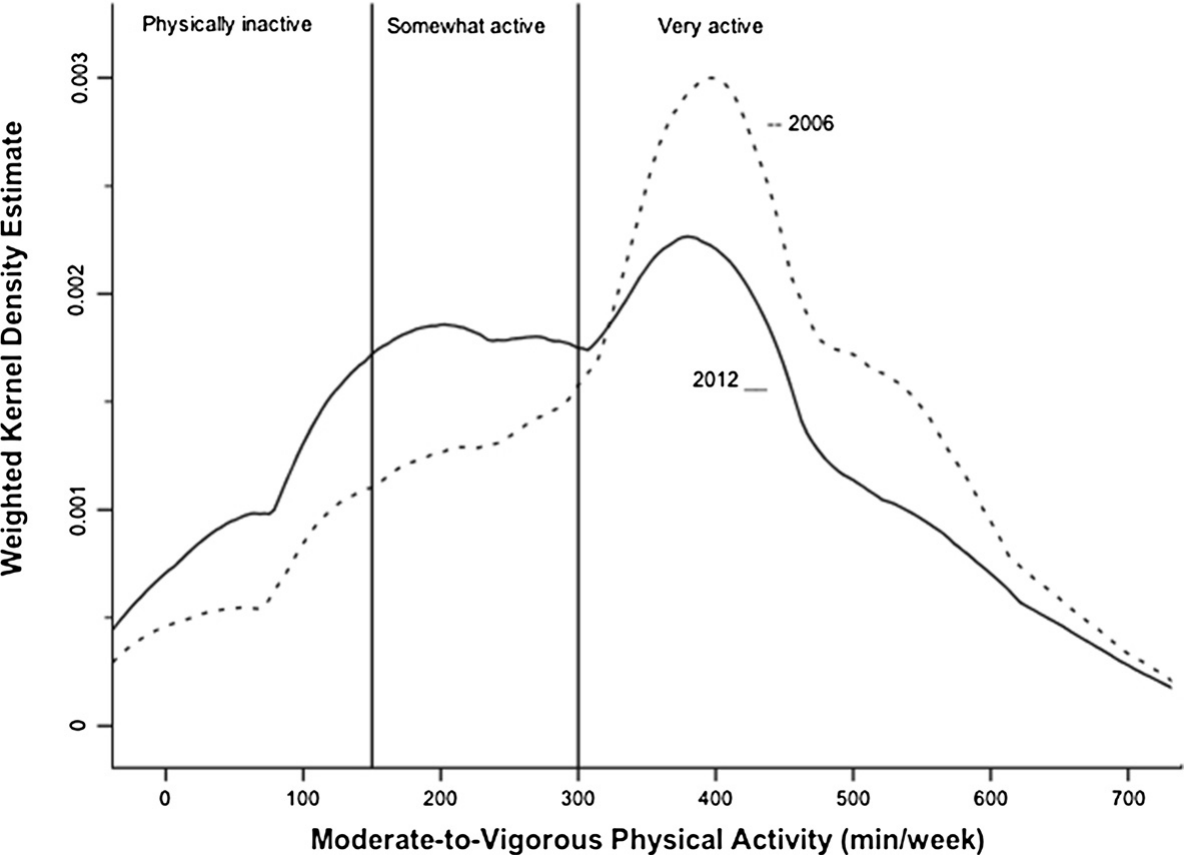
**Average moderate-to-vigorous minutes per week.** 2006–2012 ENSANUT.

The adjusted and unadjusted mean and median weekly minutes of MVPA in the 2006 and 2012 samples are described in Table 2. In 2012, the mean MVPA values were significantly lower (p < 0.01) than they were in 2006 based on both the unadjusted (897 vs. 1119 min/week) and adjusted (310 vs. 358 min/week) MVPA estimates. This observation was true in all of the sociodemographic and biological categories. Differences in the distribution of MVPA across the 2006 and 2012 samples are further illustrated in Figure 1.

**Table 2:** Mean and median moderate-to-vigorous physical activity levels (minutes per week, unadjusted and adjusted values) in 20–69 year old Mexican adults, ENSANUT 2006 and ENSANUT 2012

	**Unadjusted moderate-to-vigorous physical activity levels (minutes per week)**				**Adjusted moderate-to-vigorous physical activity levels (minutes per week)**			
	**2006**		**2012**		**2006**		**2012**	
	**N (55,137,118)**		**N (64,205,112)**		**N (55,137,118)**		**N (64,205,112)**	
	Media (SD)*	**Median (IQR) ♯**	Media (SD)*	**Median (IQR)♯**	Media (SD)*	**Median (IQR)♯**	Media (SD)*	**Median (IQR)♯**
**Total**	1119 (783)	1140 (420,1560)	897 (774)	710 (240,1350)	358 (169)	384 (230,475)	310 (172)	308 (175,415)
**Gender**								
Men	1191 (824)^b^	1200 (480,1680)	977 (831)^b^	810 (270,1440)	383 (180)^b^	389 (252,517)	338 (186)^b^	335 (188,481)
Women	1057 (741)	1080 (405,1485)	826 (711)	630 (240,1290)	337 (157)	368 (222,442)	286 (155)	287 (168,393)
*P for trend*	p < 0.001		p < 0.001		p < 0.001		p < 0.001	
**Age group**								
20-29	1100 (768)	1080 (420,1500)	882 (771)	675 (240,1320)	356 (165)	385 (237,467)	310 (171)	311 (173,411)
30-39	1149 (780)	1180 (470,1620)	901 (746)	720 (280,1350)	366 (169)	385 (252,487)	315 (166)	308 (188,415)
40-49	1177 (802)^d^	1220 (465,1650)	957 (819)	770 (250,1420)	370 (170)^d^	385 (252,496)	321 (180)	317 (178,441)
50-59	1096 (780)	1080 (420,1590)	939 (774)	780 (280,1400)	353 (172)	385 (222,475)	316 (170)	320 (184,427)
60-69	999 (785)^a,b,c,d^	910 (300,1440)	734 (725)^a,b,c,d^	480 (170,1120)	324 (172)^a,b,c,d^	336 (194,428)	268 (171)^a,b,c,d^	245 (145,386)
*P for trend*	p = 0.078		p < 0.299		p < 0.006		p < 0.299	
**BMI characteristics**								
Normal weight	1129 (772)	1155 (450,1560)	921 (755)	780 (300,1340)	362 (168)	385 (237,482)	322 (168)	314 (197,428)
Overweight	1153 (799)^c^	1180 (420,1620)	936 (798)^c^	720 (270,1400)	366 (170)	385 (237,489)	319 (173)	314 (181,432)
Obese	1077 (760)	1080 (420,1500)	850 (761)	630 (210,1305)	345 (166)^a,b^	377 (222,444)	297 (171)^a,b^	291 (161,402)
*P for trend*	p = 0.049		p < 0.027		p < 0.004		p < 0.027	
**Region of country**								
South	1216 (792)	1260 (570,1680)	1011 (802)^a,b,c^	910 (330,1456)	382 (169)	394 (272,497)	336 (178)^a,b,c^	349 (206,452)
Central	1187 (786)^c^	1260 (540,1680)	876 (774)	680 (210,1350)	372 (167)^c^	385 (263,496)	306 (177)	304 (161,415)
North	1008 (759)^b,d^	1020 (320,1460)	834 (735)	640 (220,1260)	334 (172)^b,d^	365 (206,450)	295 (170)	291 (168,402)
D.F. and metropolitan area	983 (758)^d^	900 (300,1470)	815 (745)	600 (252,1200)	325 (162)^d^	343 (206,440)	293 (153)	291 (181,391)
*P for trend*	p < 0.001		p < 0.001		p < 0.001		p < 0.001	
**Rural/urban areas**								
Rural	1264 (796)^b^	1260 (660,1680)	1053 (813)^b^	980 (360,1500)	393 (167)^b^	400 (297,504)	346 (182)^b^	366 (206,471)
Urban	1052 (768)	1050 (360,1500)	846 (754)	630 (225,1290)	342 (168)	366 (222,454)	299 (167)	295 (168,402)
*P for trend*	p < 0.001		p < 0.001		p < 0.001		p < 0.001	
**Socioeconomic status**								
Low	1253 (802)^b,c^	1260 (620,1680)	1044 (828)^b,c^	900 (360,1480)	388 (169)^b,c^	398 (288,504)	344 (181)^b,c^	356 (208,460)
Medium	1082 (766)^c^	1080 (420,1500)	882 (741)^c^	691 (245,1330)	351 (168)^c^	380 (222,460)	307 (166)^c^	303 (178,409)
High	983 (749)	920 (320,1440)	810 (746)	600 (210,1260)	327 (164)	343 (206,436)	291 (168)	291 (157,397)
*P for trend*	p < 0.001		p < 0.001		p < 0.001		p < 0.001	
**Education level**								
Primary or less	1162 (796)	1200 (450,1620)	968 (810)	820 (270,1440)	367 (172)	385 (242,496)	325 (179)	317 (181,444)
Secondary	1153(768)	1200 (480,1620)	944 (805)	780 (240,1400)	366 (165)	385 (251,486)	319 (178)	321 (175,436)
High school or higher	951 (729)^a,b^	870 (300,1410)	761 (665)^a,b^	570 (230,1180)	323 (161)^a,b^	336 (206,430)	284 (153)^a,b^	285 (173,389)
*P for trend*	p < 0.001		p < 0.001		p < 0.001		p < 0.001	

*Data presented as Mean (standard deviation) in minutes of moderate-to-vigorous physical activity per week.

♯Data presented as Median (standard deviation) in minutes of moderate-to-vigorous physical activity per week.

^a,b,c,d,e^Means with different letter are significantly different among them.

Irrespective of the survey year and whether examining the unadjusted or adjusted MVPA data, the mean weekly minutes of MVPA were significantly higher in men than in women, in the South than in the other regions of the country, and in rural areas. MVPA was lowest in the oldest age group, the highest BMI category, the highest SES level, and in the most educated group. For all categories except age groups, mean minutes per week of MVPA was significantly lower in the last categories (P for trend ≤ 0.05).

Table 3 presents the prevalence of adults who were physically inactive, sufficiently active, or very active in ENSANUT 2006 and 2012. The prevalence was calculated using both the unadjusted and adjusted physical activity data. Irrespective of whether the unadjusted or adjusted MVPA data were examined, the proportion of the adult population who were inactive significantly increased by approximately 6% between 2006 and 2012. The same significant trend was observed for gender (6% points for men and women), most age groups (5.2% points for 20–29 age group, 4.6% points for 30–39 age group, 8% points for 40–49 age group, and 9.1% points for 60–69 age group) and some BMI categories (6.2% points for overweight and 5.8% points for obese). Additional physical activity prevalence estimates are provided in Additional file 1.

**Table 3:** Prevalence and trends in inactive, sufficiently active, and very active physical activity categories in 20–69 year old Mexican adults, ENSANUT 2006 and ENSANUT 2012

**Physical activity criterion used for classification**	**Physical activity category**								
	**Inactive**			**Sufficiently active**			**Very active**		
	**2006% (95% CI)**	**2012% (95% CI)**	**Change from 2006 to 2012**	**2006% (95% CI)**	**2012% (95% CI)**	**Change from 2006 to 2012**	**2006% (95% CI)**	**2012% (95% CI)**	**Change from 2006 to 2012**
Unadjusted	11.4 (10.5,12.4)	16.0 (14.8,17.2)	4.6 (4.3,4.8)	6.5 (5.9,7.1)	10.9 (9.8,12.0)	4.4 (3.9,4.9)	82.1 (81.0,83.2)	73.2 (71.6,74.6)	-8.9 (-9.4,-8.6)
Adjusted	13.4 (12.5, 14.5)	19.4 (18.1, 20.7)	6.0 (5.6,6.2)	19.7 (18.6, 20.8)	28.8 (27.2, 30.5)	9.1 (8.6,9.7)	66.9 (65.5, 68.3)	51.8 (50.0, 53.5)	-15.1 (-15.5,-14.8)

Table 4 provides the results for the multivariate logistic regression models that were used to evaluate the association between physical inactivity with the sociodemographic and biological variables. Based on the multivariate model for the adjusted MVPA data, the oldest age group, having obesity, or being in the highest SES level were significantly associated with physical inactivity.

**Table 4:** Association between sociodemographic characteristics and being physically inactive (<150 min/week of moderate-to-vigorous physical activity) in 20–69 year old Mexican adults based on unadjusted and adjusted physical activity levels, ENSANUT 2012

	***Unadjusted *** **moderate-to-vigorous physical activity levels**		***Adjusted *** **moderate-to-vigorous physical activity levels**	
	**Bivariate model**	**Multivariate model**	**Bivariate model**	**Multivariate model**
	OR (CI 95%)*	**OR (CI 95%)♯**	OR (CI 95%)*	**OR (CI 95%)♯**
**Gender**				
Men	1	1	1	1
Women	1.31 (1.02,1.66)	1.24 (0.96,1.60)	1.18 (1.00,1.40)	1.14 (0.95,1.37)
**Age group**				
20-29	1	1	1	1
30-39	0.83 (0.59,1.15)	0.73 (0.51,1.04)	0.93 (0.72,1.22)	0.88 (0.68,1.16)
40-49	0.96 (0.69,1.33)	0.80 (0.56,1.15)	1.11 (0.86,1.43)	1.05 (0.80,1.38)
50-59	0.95 (0.66,1.38)	0.79 (0.53,1.18)	1.04 (0.80,1.35)	0.96 (0.72,1.27)
60-69	1.35 (0.83,2.21)	1.20 (0.71,2.03)	1.59 (1.20,2.11)	1.58 (1.15,2.17)
**BMI Classification**				
Normal weight	1	1	1	1
Overweight	1.20 (0.88,1.65)	1.22 (0.90,1.65)	1.14 (0.89,1.44)	1.10 (0.86,1.39)
Obese	1.46 (1.08,2.00)	1.43 (1.05,1.96)	1.38 (1.08,1.75)	1.30 (1.01,1.65)
**Region of country**				
South	1	1	1	1
Central	1.58 (1.23,2.03)	1.56 (1.19, 2.03)	1.29 (1.07,1.54)	1.19 (0.97,1.46)
North	1.29 (1.02, 1.65)	1.19 (0.91,1.54)	1.31 (1.10,1.56)	1.14 (0.94, 1.39)
D.F. and metropolitan area	1.32 (0.87,2.03)	1.09 (0.70,1.71)	0.99 (0.70,1.39)	0.83 (0.57,1.20)
**Rural/urban areas**				
Rural	1	1	1	1
Urban	1.66 (1.32,2.09)	1.59 (1.21,2.09)	1.26 (1.05,1.50)	1.07 (0.88,1.31)
**Socioeconomic status**				
Low	1	1	1	1
Medium	1.44 (1.07,1.93)	1.28 (0.92,1.77)	1.31 (1.04,1.65)	1.30 (1.01,1.67)
High	1.47 (1.12,1.94)	1.18 (0.83,1.69)	1.61 (1.31,1.99)	1.60 (1.21,2.09)
**Education level**				
Primary or less	1	1	1	1
Secondary	0.92 (0.67,1.27)	0.88 (0.62,1.26)	1.09 (0.88,1.36)	1.14 (0.90, 1.46)
High school or higher	0.94 (0.66,1.35)	0.83 (0.55,1.24)	1.12 (0.88,1.43)	1.07 (0.79, 1.45)

*Odds ratios (95% confidence interval) for being inactive. Odds ratios are not adjusted.

♯Odds ratios (95% confidence interval) for being physically inactive. Odds ratios are adjusted for all of the other sociodemographic variables listed in the first column of the table.

## Discussion

The objectives of this study were to describe the prevalence of physical inactivity in Mexican adults, to examine changes in the prevalence of physical inactivity between 2006 and 2012, and to examine the association between physical inactivity with several sociodemographic and biological characteristics. The adjusted physical activity estimate indicates that 19.4% of the adult Mexican population was physically inactive in 2012. This represented an absolute increase of 6.0%, or a relative increase of 44%, since 2006. At the same time, 28.8% of the adult population was sufficiently active in 2012 (e.g., met minimal physical activity recommendations), and 51.8% were considered to be very active. Finally, MVPA levels varied considerably according to gender, age, BMI, region of the country, SES, and education level.

Based on unadjusted IPAQ data from the ENSANUT 2006, Gomez et al. previously reported that 11% of the adult population in Mexico did not meet physical activity recommendations [20]. Outside of Mexico, adherence to physical activity recommendations has been reported for 121 other countries, as recently summarized by Hallal et al. [1]. These authors estimated that the global prevalence of physical inactivity in adults is 31%, with a range of 5% (Bangladesh) to 72% (Malta). For the Mexican population, they reported a physical inactivity prevalence of 37.7%, which is 18.3 points higher than the value we reported in our article (19.4%). Potential reasons for this discrepancy includes the use of different physical activity questionnaires and difference in the physical activity and inactivity were classified.

Although the number of minutes of MVPA was significantly different between men and women, the probability of being physically inactive was not significantly lower in women after adjusting for sociodemographic variables. This result is in line with what has been found in other Latin American countries [21].

We observed that 60–69 year olds were approximately 1.58 times more likely to be physically inactive than 20–29 year olds. This finding is consistent with age differences in MVPA that have been described globally [1,5]. Conversely, while SES is negatively associated with physical inactivity in developed countries [22], the opposite pattern has been observed in Mexican adults in our study and previous Mexican research [20,23]. Thus, Mexican adults within the highest SES group were significantly less likely to engage in recommended levels of MVPA. A potential explanation for the SES gradient observed in Mexico is that people with a lower SES have the most physically demanding jobs, and due to a lack of resources do not have access to motorized transportation.

As noted previously in ENSANUT 2006 [20] and in our study for ENSANUT 2012, obese adults are less active than adults with a BMI in the healthy range. In fact, in our study obese individuals were 1.30 times more likely to be physically inactive. Obesity is an independent risk factor for several chronic diseases such as type 2 diabetes, cardiovascular disease, stroke, and several cancers [19,24]. Thus, part of the pathway through which physical inactivity influence non-communicable disease risk is by contributing to obesity. However, it is important to note that physical inactivity has an effect on non-communicable chronic disease risk that is independent of its effects on obesity [2]. Regardless of the pathways by which physical inactivity influences health, it is clearly an important risk factor for non-communicable disease within the Mexican population. Recently, Lee et al. [2] estimated that 6% of coronary heart disease cases and 8% of type 2 diabetes cases in the Mexican population are directly attributable to physical inactivity [2]. Additionally, they estimated that life expectancy in Mexican adults would increase by 0.76 years if physical inactivity was eliminated [2]. Taken together, these findings highlight the importance of having policies and programs in place within Mexico to increase the physical activity levels of the population [25].

The differences between the unadjusted and adjusted MVPA data were quite striking. For instance, the mean MVPA in ENSANUT 2012 was 897 min/week before being adjusted and 310 min/week after being adjusted. We felt that it was important to provide adjusted estimates because several previous studies have reported that self-reported measures of MVPA are greatly overestimated [13,14]. This was confirmed by our validity study in Mexican population [11]. We believe that the adjusted physical activity estimates are a closer approximation of the true physical activity levels of the population and recommend that the adjusted data be used in future papers and reports that present data on the physical inactivity levels of Mexicans obtained using the IPAQ short.

An important limitation of this study is that the physical activity data were self-reported. We attempted to correct for the over-reporting by adjusting the self-reported IPAQ with the developed equation [11]. However, such an adjustment is far from perfect as self-reported and accelerometer measures of MVPA are only modestly correlated [10,11,26-28]. This adjustment has the disadvantage to enclose moderate and vigorous activity into a single measure. Although moderate and vigorous activities are not separable with this measure, it was appropriate for estimating prevalence of physical inactivity at the given cut-points. In addition, the validity study was conducted on a reasonably small (n = 262) sample of employed adults from Mexico City and the generalizability of the findings from that sample to the more diverse ENSANUT sample is unknown.

Another important limitation is the use of the short form IPAQ to estimate physical activity levels. Although it is the most commonly used instrument within large-scale surveys, this instrument overestimates physical activity when compared to other questionnaires [29] and underestimates physical activity levels when compared to long form IPAQ [30]. This underestimation could be related to the fact that the short form IPAQ does not measure the different physical activity domains (e.g., recreational physical activity, inside home activities, transportation) [31].

## Conclusions

In conclusion, while the average Mexican adult engaged in approximately 300 minutes per week of MVPA, 19.4% do not meet the minimal recommendation of 150 minutes per week. The prevalence of the population not meeting this recommendation has increased by 6% points over the past 6 years. The groups at higher risk were those older than 60 years old, obese persons, and individuals within the highest socioeconomic tertile. Public health interventions should be focused on increasing physical activity levels, specifically in those disadvantaged groups.

## Competing interests

The authors declare that they have no competing interests.

## Authors’ contributions

MC carried out the study design, data collection manual, training of field personnel, and drafted the manuscript. BS contributed to the study design, analysis and discussion. CI participated in the statistical analysis and preparation of the manuscript. JI conceived of the study with MC and BS, and participated in its design and helped to draft the manuscript. All authors read and approved the final manuscript.

## Pre-publication history

The pre-publication history for this paper can be accessed here:

http://www.biomedcentral.com/1471-2458/13/1063/prepub

## Supplementary Material

Additional file 1: Table S1 Prevalence in inactive, sufficiently active, and very active physical activity categories in 20-69 year old Mexican adults based on "unadjusted" physical activity data, ENSANUT 2006 and ENSANUT 2012. **Table S2.** Prevalence in inactive, sufficiently active, and very active physical activity categories in 20–69 year old Mexican adults based on “adjusted” physical activity data, ENSANUT 2006 and ENSANUT 2012. Click here for file
