# Supplementary material for: Physical inactivity prevalence and trends among Mexican adults: results from the National Health and Nutrition Survey (ENSANUT) 2006 and 2012
Source: BMC Public Health. 2013 Nov 11;13:1063. doi: 10.1186/1471-2458-13-1063 (PMC3883516; doi:10.1186/1471-2458-13-1063)
Supplement: Additional file 1: Table S1 — Prevalence in inactive, sufficiently active, and very active physical activity categories in 20-69 year old Mexican adults based on "unadjusted" physical activity data, ENSANUT 2006 and ENSANUT 2012. Table S2. Prevalence in inactive, sufficiently active, and very active physical activity categories in 20–69 year old Mexican adults based on “adjusted” physical activity data, ENSANUT 2006 and ENSANUT 2012. [file 1471-2458-13-1063-S1.doc]

| **Appendix 1 - Table 1. Prevalence in inactive, sufficiently active, and very active physical activity categories in 20-69 year old Mexican adults based on "*unadjusted*" physical activity data, ENSANUT 2006 and ENSANUT 2012** | | | | | | | | | |
| --- | --- | --- | --- | --- | --- | --- | --- | --- | --- |
|  |  | **Physical Activity Category** | | | | | | | |
|  |  | **Inactive** | |  | **Sufficiently Active** | |  | **Very Active** | |
|  |  | 2006 | 2012 |  | 2006 | 2012 |  | 2006 | 2012 |
| **Gender** |  |  |  |  |  |  |  |  |  |
| Men |  | 10.5 (9.4,11.8) | 15.2 (13.3,17.3) |  | 5.5 (4.7,6.4) | 9.6 (8.1,11.5) |  | 84.0 (82.5,85.4) | 75.2 (72.7,77.5) |
| Women |  | 12.1 (10.9,13.4) | 16.7 (15.1,18.3) |  | 7.4 (6.6,8.3) | 12.0 (10.6,13.5) |  | 80.5 (79.0,81.9) | 71.4 (69.4,73.3) |
| **Age group** |  |  |  |  |  |  |  |  |  |
| 20-29 |  | 10.7 (9.0,12.7) | 15.8 (13.5,18.3) |  | 7.0 (5.7,8.5) | 11.2 (9.1,13.8) |  | 82.3 (80.1,84.3) | 73.0 (70.0,75.9) |
| 30-39 |  | 11.0 (9.6,12.7) | 14.8 (12.2,17.8) |  | 5.8 (4.9,7.0) | 9.6 (7.8,11.7) |  | 83.1 (81.2,84.9) | 75.6 (72.3,78.6) |
| 40-49 |  | 10.7 (8.9,12.7) | 16.0 (13.6,18.8) |  | 6.1 (5.0,7.3) | 10.8 (8.8,13.1) |  | 83.3 (81.0,85.3) | 73.2 (70.0,76.2) |
| 50-59 |  | 12.0 (10.3,14.1) | 14.3 (11.7,17.3) |  | 6.1 (4.8,7.8) | 10.9 (8.5,13.9) |  | 81.8 (79.5,84.0) | 74.8 (71.1,78.1) |
| 60-69 |  | 15.0 (12.8,17.5) | 22.0 (18.4,26.1) |  | 8.4 (6.6,10.7) | 13.4 (9.3,26.1) |  | 76.6 (73.5,79.4) | 64.6 (59.4,69.4) |
| **BMI Classification** |  |  |  |  |  |  |  |  |  |
| Normal weight |  | 10.2 (8.7,12.0) | 13.9 (11.6,16.4) |  | 6.6 (5.5,8.0) | 9.3 (7.6,11.4) |  | 83.1 (81.0,85.0) | 76.9 (73.9,79.6) |
| Overweight |  | 10.6 (9.4,11.8) | 14.7 (13.0,16.7) |  | 6.7 (5.8,7.8) | 10.8 (9.0,13.0) |  | 82.7 (81.2,84.2) | 74.4 (71.9,76.9) |
| Obese |  | 13.1 (11.5,15.0) | 17.7 (15.4,20.1) |  | 6.3 (5.4,7.4) | 12.4 (10.4,14.7) |  | 80.5 (78.5,82.4) | 69.9 (66.9,72.8) |
| **Region of country** |  |  |  |  |  |  |  |  |  |
| South |  | 8.9 (7.7,10.3) | 14.3 (12.7,16.0) |  | 5.4 (4.8,6.1) | 8.9 (7.6,10.3) |  | 85.7 (84.1,87.1) | 76.8 (74.7,78.8) |
| Central |  | 9.9 (8.8,11.2) | 16.9 (15.0,18.9) |  | 5.5 (4.7,6.4) | 12.9 (11.0,15.0) |  | 84.6 (83.1,85.9) | 70.3 (67.7,72.7) |
| North |  | 15.0 (13.2,17.0) | 18.2 (16.5,20.2) |  | 8.3 (7.0,9.7) | 10.6 (9.2,12.3) |  | 76.8 (74.4,79.0) | 71.1 (68.9,73.3) |
| D.F. and metropolitan areas |  | 13.7 (10.7,17.4) | 14.9 (10.9,19.9) |  | 7.9 (6.0,10.4) | 11.3 (10.9,19.9) |  | 78.4 (74.5,81.8) | 73.8 (68.4,78.6) |
| **Rural/urban areas** |  |  |  |  |  |  |  |  |  |
| Rural |  | 7.9 (6.9,9.0) | 14.0 (12.2,16.0) |  | 4.9 (4.3,5.7) | 7.8 (6.5,9.3) |  | 87.2 (85.8,88.4) | 78.2 (75.9,80.3) |
| Urban |  | 13.0 (11.8,14.3) | 16.6 (15.1,18.2) |  | 7.2 (6.4,8.1) | 11.9 (10.6,13.3) |  | 79.8 (78.3,81.2) | 71.5 (69.7,73.3) |
| **SES** |  |  |  |  |  |  |  |  |  |
| Low |  | 9.0 (8.0,10.1) | 12.9 (11.2,14.8) |  | 4.9 (4.3,5.6) | 8.8 (7.3,10.5) |  | 86.1 (84.9,87.3) | 78.3 (75.9,80.6) |
| Medium |  | 11.8 (10.4,13.3) | 14.7 (12.9,16.7) |  | 6.9 (5.8,8.2) | 11.9 (9.9,14.2) |  | 81.3 (79.5,83.0) | 73.4 (70.6,76.1) |
| High |  | 14.0 (12.0,16.4) | 19.0 (16.9,21.2) |  | 8.2 (6.9,9.7) | 11.5 (9.7,13.6) |  | 77.8 (75.3,80.1) | 69.5 (66.9,72.0) |
| **Education level** |  |  |  |  |  |  |  |  |  |
| Primary or less |  | 11.3 (10.1,12.6) | 13.7 (12.0,15.7) |  | 6.0 (5.2,6.8) | 11.7 (9.2,14.8) |  | 82.7 (81.2,84.1) | 74.6 (71.6,77.4) |
| Secondary |  | 10.3 (8.8,12.1) | 16.1 (14.3,18.2) |  | 5.8 (4.6,7.3) | 10.7 (9.2,12.2) |  | 83.9 (81.7,85.9) | 73.2 (70.9,75.4) |
| High school or higher |  | 13.5 (11.2,16.1) | 17.3 (15.0,19.9) |  | 8.6 (7.1, 10.4) | 10.7 (8.8,12.9) |  | 78.0 (75.1,80.6) | 72.0 (69.1,74.8) |
| Data are presented as a % (95% confidence interval) | | | | | | | | | |

| **Appendix 1 - Table 2. Prevalence in inactive, sufficiently active, and very active physical activity categories in 20-69 year old Mexican adults based on "*adjusted*" physical activity data, ENSANUT 2006 and ENSANUT 2012** | | | | | | | | | |
| --- | --- | --- | --- | --- | --- | --- | --- | --- | --- |
|  |  | **Physical Activity Category** | | | | | | | |
|  |  | **Inactive** | |  | **Sufficiently Active** | |  | **Very Active** | |
|  |  | 2006 | 2012 |  | 2006 | 2012 |  | 2006 | 2012 |
| **Gender** |  |  |  |  |  |  |  |  |  |
| Men |  | 12.0 (10.8, 13.4) | 18.0 (16.1, 20.2) |  | 17.6 (16.0, 19.4) | 25.1 (22.7, 27.5) |  | 70.3 (68.2, 72.3) | 56.9 (54.2, 59.5) |
| Women |  | 14.6 (13.3, 16.0) | 20.6 (19.0, 22.3) |  | 21.4 (20.1, 22.7) | 32.1 (29.9, 34.5) |  | 64.0 (62.3, 65.6) | 47.3 (44.9, 49.6) |
| **Age group** |  |  |  |  |  |  |  |  |  |
| 20-29 |  | 13.2 (11.4, 15.2) | 18.4 (16.1, 21.1) |  | 19.5 (17.3, 21.9) | 30.0 (26.5, 33.8) |  | 67.3 (64.6, 69.9) | 51.5 (48.0, 55.1) |
| 30-39 |  | 12.8 (11.3, 14.5) | 17.4 (14.8, 20.5) |  | 18.8 (17.1, 20.7) | 29.4 (26.2, 32.9) |  | 68.3 (66.0, 70.6) | 53.2 (49.3, 57.0) |
| 40-49 |  | 12.1 (10.3, 14.3) | 20.1 (17.4, 23.0) |  | 18.9 (16.8, 21.2) | 25.9 (22.9, 29.1) |  | 69.0 (66.4, 71.4) | 54.1 (50.6, 57.5) |
| 50-59 |  | 14.4 (12.4, 16.7) | 19.1 (16.1, 22.4) |  | 20.4 (18.0, 23.0) | 27.0 (23.0, 31.4) |  | 65.2 (62.2, 68.1) | 53.9 (49.2, 58.6) |
| 60-69 |  | 17.3 (14.9, 20.0) | 26.4 (22.3, 31.0) |  | 22.9 (19.6, 26.5) | 33.0 (27.6, 38.9) |  | 59.8 (56.0, 63.6) | 40.6 (36.1, 45.2) |
| **BMI Classification** |  |  |  |  |  |  |  |  |  |
| Normal weight |  | 12.5 (10.7, 14.4) | 16.6 (14.2, 19.2) |  | 19.9 (17.9, 22.2) | 29.3 (26.1, 32.7) |  | 67.6 (65.1, 70.0) | 54.1 (50.8, 57.4) |
| Overweight |  | 12.2 (11.0, 13.6) | 18.4 (16.2, 20.8) |  | 20.0 (18.5, 21.7) | 27.2 (24.8, 29.8) |  | 67.7 (65.7, 69.7) | 54.4 (51.5, 57.2) |
| Obese |  | 15.5 (13.7, 17.5) | 21.4 (19.1, 24.0) |  | 18.8 (17.2, 20.5) | 30.6 (27.7, 33.6) |  | 65.7 (63.4, 67.9) | 48.0 (44.8, 51.2) |
| **Region of country** |  |  |  |  |  |  |  |  |  |
| South |  | 10.9 (9.6, 12.5) | 17.4 (23.9, 21.8) |  | 16.9 (15.8, 18.0) | 23.9 (21.8, 26.2) |  | 72.2 (70.4, 73.9) | 58.6 (56.0, 61.2) |
| Central |  | 11.5 (10.3, 12.8) | 21.3 (19.2, 23.7) |  | 17.9 (16.3, 19.6) | 27.7 (25.0, 30.6) |  | 70.7 (68.7, 72.6) | 50.9 (48.2, 53.7) |
| North |  | 17.5 (15.7, 19.6) | 21.7 (19.8, 23.7) |  | 20.9 (19.1, 22.7) | 29.8 (27.4, 32.2) |  | 61.6 (58.9, 64.2) | 48.6 (46.0, 51.1) |
| D.F. and metropolitan areas |  | 16.0 (12.9, 19.8) | 17.3 (13.2, 22.3) |  | 25.3 (21.7, 29.3) | 37.0 (31.6, 42.9) |  | 58.6 (54.0, 63.1) | 45.7 (39.9, 51.6) |
| **Rural/urban areas** |  |  |  |  |  |  |  |  |  |
| Rural |  | 9.6 (8.4, 10.9) | 16.8 (14.9, 19.0) |  | 15.6 (14.3, 17.0) | 21.5 (19.4, 23.9) |  | 74.8 (73.0, 76.4) | 61.7 (59.1, 64.2) |
| Urban |  | 15.2 (13.9, 16.6) | 20.2 (18.7, 21.9) |  | 21.5 (20.1, 22.9) | 31.2 (29.2, 33.2) |  | 63.3 (61.5, 65.1) | 48.6 (46.4, 50.7) |
| **SES** |  |  |  |  |  |  |  |  |  |
| Low |  | 10.8 (9.7, 12.0) | 15.2 (13.3,17.4) |  | 15.5 (14.4, 16.7) | 24.6 (22.2, 27.1) |  | 73.7 (72.2, 75.1) | 60.2 (57.5, 62.9) |
| Medium |  | 14.1 (12.6, 15.6) | 19.0 (16.7, 21.6) |  | 20.1 (18.3, 22.0) | 30.1 (27.3, 33.2) |  | 65.8 (63.7, 68.0) | 50.8 (47.8, 53.9) |
| High |  | 16.1 (14.0, 18.5) | 22.5 (20.3, 24.8) |  | 24.8 (22.6, 27.1) | 30.6 (27.7, 33.7) |  | 59.1 (56.2, 62.0) | 46.9 (43.8, 50.0) |
| **Education level** |  |  |  |  |  |  |  |  |  |
| Primary or less |  | 13.4 (12.1, 14.8) | 18.2 (15.7, 21.0) |  | 17.8 (16.5, 19.1) | 26.6 (23.0, 30.5) |  | 68.8 (67.1, 70.5) | 55.2 (51.6, 58.7) |
| Secondary |  | 12.0 (10.2, 14.0) | 19.6 (17.7, 21.7) |  | 18.8 (16.7, 21.1) | 26.6 (24.5, 28.9) |  | 69.2 (66.6, 71.7) | 53.8 (51.1, 56.4) |
| High school or higher |  | 15.8 (13.5, 18.5) | 19.9 (17.6, 22.6) |  | 25.4 (22.7, 28.4) | 34.4 (30.9, 38.0) |  | 58.7 (55.6, 61.8) | 45.7 (42.1, 49.3) |
| Data are presented as a % (95% confidence interval) | | | | | | | | | |
